# Multisystem inflammatory syndrome in children (MIS-C) post-COVID-19 in Iran: clinical profile, cardiac features, and outcomes

**DOI:** 10.1186/s12887-024-04652-y

**Published:** 2024-03-13

**Authors:** Ali Hoseininasab, Reza Sinaei, Mohammad Mehdi Bagheri, Maryam Ahmadipour, Reza Derakhshan, Mohammad Javad Najafzadeh, Fatemeh Karami Robati, Maedeh Jafari, Sarehossadat Ebrahimi, Mohammad Ali Jafari

**Affiliations:** 1 Research Center of Tropical and Infectious Diseases, Kerman University of Medical Sciences, Kerman, Iran; 2 Department of Pediatrics, School of Medicine, Kerman University of Medical Sciences, Kerman, Iran; 3 Endocrinology and Metabolism Research Center, Institute of Basic and Clinical Physiology Sciences, Kerman University of Medical Sciences, Kerman, Iran; 4 Cardiovascular Research Center, Institute of Basic and Clinical Physiology Sciences, Kerman University of Medical Sciences, Kerman, Iran; 5 Department of Pediatrics, Afzalipour Faculty of Medicine, Kerman University of Medical Sciences, Kerman, Iran; 6 School of Medicine, Kerman University of Medical Sciences, Kerman, Iran; 7 Clinical Research Development Unit, Afzalipour Hospital, Kerman University of Medical Sciences, Kerman, Iran; 8 Department of Veterinary Basic Sciences, Science and Research Branch, Islamic Azad University, Tehran, Iran

**Keywords:** Pericardial effusion, Pediatrics, COVID-19, Pediatric multisystem inflammatory disease

## Abstract

**Background:**

In April 2020, an association between multisystem inflammatory syndromes (MIS-C) was observed in children with severe acute respiratory syndrome coronavirus infection (SARS-CoV-2). Most patients had heart involvement alone, and most patients had pericardial effusion. This study aimed to express and emphasize cardiac involvement in pediatric patients with respiratory symptoms who were diagnosed with COVID-19.

**Methods:**

This study was conducted in July 2021 in Kerman province, Southeastern Iran, during a notable surge in severe acute respiratory syndrome coronavirus 2 (SARS-CoV-2) infections. The study included 904 pediatric patients diagnosed with COVID-19. Data collection involved a comprehensive assessment of clinical symptoms and manifestations. Patients with fever lasting more than five days were admitted to the hospital. Echocardiography was utilized for cardiac involvement diagnosis, with 47 patients undergoing this diagnostic procedure.

**Results:**

Of the 904 patients, most of them had high fevers (74%). Fifty-five patients had a fever for more than five days and were hospitalized. Of the 47 patients who underwent echocardiography, 45 (81%) had heart involvement. In 75% of patients, pericardial effusion was the only cardiac involvement. Patients with pericardial effusion were treated with dexamethasone up to 3 mg every 8 h for 72 h.

**Conclusions:**

MIS-C has a wide range of clinical symptoms. In cases where the fever is prolonged and there are gastrointestinal symptoms, physicians have clinical suspicion to diagnose this syndrome. Most cases of pericardial effusion are alone and improve with treatment with glucocorticosteroids.

## Background

The coronavirus disease 2019 (COVID*-*19) pandemic emerged in 2019, originating from Wuhan, China, and rapidly spread across the globe. By April 2020, an association between multisystem inflammatory syndromes (MIS-C) and severe acute respiratory syndrome coronavirus infection (SARS-CoV-2) had been noted, particularly among children and adolescents [1, 2]. This syndrome shares several similarities with Kawasaki disease [3]. MIS-C is a recognized complication of coronavirus infection in children and has been extensively discussed in scientific literature [3, 4]. Characterized by nonspecific symptoms such as fever and gastrointestinal manifestations, MIS-C involves multiple organ systems, as outlined by the World Health Organization (WHO) [5].

Studies by Dufort et al. reported an incidence of two cases per 100,000 for MIS-C [6]. Lee et al. investigated demographic factors, including age, sex, and MIS-C incidence in New York City [7]. The Verdoni study highlighted clinical signs of MIS-C, emphasizing cardiac involvement in patients, with heart failure, shock, and pericardial effusion being the most prevalent cardiac manifestations [8].

We investigated a cohort of 904 pediatric patients with respiratory symptoms, all diagnosed with COVID-19. This study aims to explore demographic characteristics, treatment modalities, and outcomes among patients diagnosed with MIS-C. Notably, most patients exhibited isolated heart involvement, particularly with pericardial effusion, emphasizing cardiac implications.

## Methods

This cross-sectional study was conducted in July 2021 in Kerman province, Southeastern Iran, during a notable surge in severe acute respiratory syndrome coronavirus 2 (SARS-CoV-2) infections. During the fifth peak of COVID-19 in Iran on July 5, 2021, a group of children was infected with the SARS-CoV-2 Delta serotype. Within this period, 904 pediatric patients were diagnosed with COVID-19 by specialized pediatric infectious diseases physicians in Kerman province, Southeastern Iran. Patients exhibiting clinical symptoms compatible with COVID-19, alongside a positive SARS-CoV-2 PCR test or with confirmed exposure to family members diagnosed with COVID-19, were included in this study. Specifically, patients presenting with fever and displaying symptoms involving the gastrointestinal and respiratory systems were enrolled. Diagnosis of Multisystem Inflammatory Syndrome in Children (MIS-C) was based on the criteria set forth by the World Health Organization (WHO); these descriptions share standard components, including persistent fever, dysfunction in multiple organs, heightened levels of inflammatory markers, and recent or ongoing exposure to SARS-CoV-2 infection [5].

Ethical approval for the study was obtained from the Organizational Ethics Committee before commencement (IR.KMU.AH.REC.1400.126).

During the study period, the census method included all the children with the conditions to enter the study. The patient information was collected prospectively through history taking, physical examination, and laboratory assessments. Specifically, a dedicated team of trained healthcare professionals, including physicians and nurses, collected the data. They interacted directly with the patients and their families to obtain detailed information on disease manifestations, familial disease history, laboratory findings, cardiac assessments, and preferred treatment modalities. Data were recorded and organized using Excel spreadsheets. Hospitalization was recommended for patients experiencing fever lasting more than five days. Those exhibiting prolonged fever, an Erythrocyte Sedimentation Rate (ESR) exceeding 40 mm/h, and C-reactive protein (CRP) levels above 30 mg/dl underwent echocardiography. Laboratory findings were interpreted against established standard ranges.

Pediatric cardiologists performed echocardiography. On the parasternal peripheral view using two-dimensional imaging, a left ventricular posterior wall fluid measurement of more than 2 mm indicates pericardial effusion. Ventricular failure was determined by M-mode and Simson methods with values less than 54% for left ventricular ejection fraction or less than 28% for fractional shortening. The color Doppler method was used to identify atrioventricular valve insufficiency according to its presence if the insufficiency was more than one square millimeter. Coronary artery dilatation was evaluated more than 1.5 times the average diameter and was compared with the adjacent normal sections using the Z-score of the coronary artery. Echocardiographic results were evaluated using the extracted Z values per established references [9].

Additionally, patients diagnosed with pericardial effusion were treated with glucocorticoids, specifically dexamethasone, administered up to 3 mg every 8 h for 72 h. Conversely, patients identified with coronary artery aneurysms, with or without concurrent pericardial effusion, received Intravenous Immunoglobulin (IVIG) therapy. This therapeutic approach aligned with established protocols to manage MIS-C-related complications involving cardiac manifestations [10].

Demographic characteristics, including age and gender, were analyzed using appropriate statistical tests such as the independent t-test, Mann-Whitney U test, chi-square test, or Fisher’s exact test. Clinical manifestations, covering various symptoms, were compared using chi-square tests or Fisher’s exact tests for categorical variables, and mean age differences among symptom groups were assessed using the independent t-test or ANOVA. Hospitalization-related echocardiography rates were compared using chi-square or Fisher’s exact tests. Laboratory findings, including lymphopenia and inflammatory markers (CRP, ESR), underwent analysis with chi-square tests, independent t-tests, or Mann-Whitney U tests. Incidence rates of pericardial effusion and coronary artery aneurysms were compared using chi-square tests or Fisher’s exact tests. Outcomes and symptom severity were assessed via chi-square or Fisher’s exact tests. SPSS22 was used for these analyses, with a significance level 0.05 for all tests.

## Results

The mean age of children was approximately 14 ± 3.7 years, with 72% being boys.

Most children impacted in this study (95%, 856) either experienced mild COVID-19 symptoms within the past six weeks or had a family member confirmed to have COVID-19 despite the child not exhibiting evident clinical symptoms.

Among the patients assessed, a majority (74%, 671) presented with high fever, while 38.2% (345) exhibited symptoms of coryza. Gastrointestinal symptoms were observed in 58% (76) with diarrhea, 54% (70) with vomiting, and 31.16% (282) with cough. Several patients also had a sore throat (10.1%, 91) and skin rashes (1.58%, 14) (Table 1).

**Table 1:** Epidemiologic characteristics, clinical features, hospitalization, echocardiography findings, treatment modality, and outcome of 904 children with suspected SARS-CoV-2 infection

Characteristic	Value
Age Median (range)	168 (3-206) mo
Age Group Distribution — no. (%)	
<1 yr	81 (9)
1–5 yr	416 (46)
6–10 yr	271 (30)
11–15 yr	136 (15)
Sex — no. (%)	
Male	650 (72)
Female	254 (28)
Diagnosis information — no. (%)	
Confirmed Cases	138 (15%)
Confirmed family members	904 (100%)
Fever — no. (%)	671 (74)
Median duration of fever (range) — days	3 (1–11)
Diarrhea — no. (%)	70 (7. 7)
Sore throat— no. (%)	91 (10.1)
Coriza symptoms— no. (%)	345 (38.2)
Abdominal pain— no. (%)	104 (11.5)
Vomiting— no. (%)	70 (7.75
Skin rash— no. (%)	14 (1.5)
Bacterial Superinfection	
AOM	20 (2.2)
ABRS	40 (4.4)
Abnormalities on echocardiography Number. (%)	
Mitral regurgitation	21 (44.4)
Mild Pericardial effusion	35 (75.6)
Tricuspid regurgitation	17 (37.8)
Coronary aneurysm	9 (20)
Myocarditis	6 (13.3)
Duration of hospitalization Median (range) day	5.7 (2–13)
Treatment	
IVIG	12 (21.8)
Glucocorticoid	41 (74.5)
ICU Care— no. (%)	7 (12.7)
Intubation— no. (%)	2 (3.6)
Death	2 (3.6)
Remission	53 (96.4)

AOM: Acute Otitis Media, ABRS: Acute Bacterial Rhinosinusitis

Notably, twenty patients were diagnosed with acute otitis media, and forty presented with acute bacterial sinusitis, all of whom received appropriate antibiotic therapy.

**Fig. 1:**
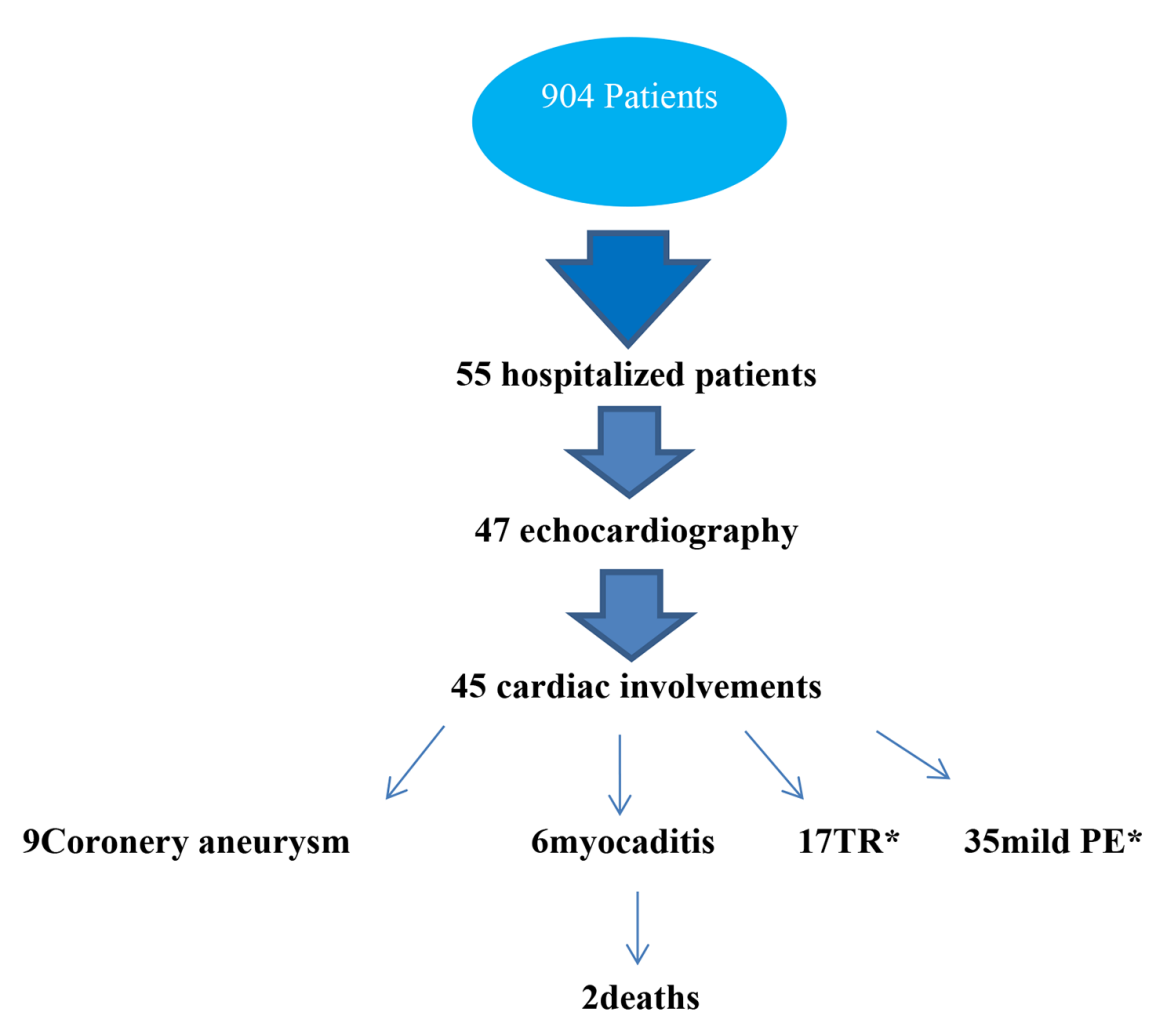
Algorithm of hospitalized patients and cardiac complications (PE accompaniment in 10TR, 4myocarditis, 8coronery aneurysm) (*PE: pericardial effusion, tricuspid regurgitation)

Fifty-five patients with a fever lasting more than five days and an ill appearance were admitted and investigated in the hospital (Fig. 1). All these cases had elevated inflammatory markers and changes in some other laboratory tests. Among them, 47 underwent echocardiography, revealing heart involvement in 45 patients (81%). Laboratory findings have been compared in children with different echocardiographic findings. Patients with coronary artery involvement had higher ESR and CRP than the group without coronary artery involvement (Table 2).

**Table 2:** Comparison of laboratory findings in patients with and without coronary artery involvement

Laboratory test	All cardiac involvement (*N* = 47)	Abnormal coronary artery (*N* = 9)	Normal coronary artery (*N* = 38)	*P* Value
WBC (×10^3^ /mm^3^)	12.25 ± 19.11	13.97 ± 10.011	10.37 ± 9.013	0.343
Lymphocyte (×10^3^ /mm^3^)	5.145 ± 6.12	1.134 ± 1.004	4.215 ± 5.114	0.232
Hb (mg/dl)	11.1 ± 2.33	9.78 ± 2.54)	10.57 ± 1.25	0.466
ESR (mm/h)	50.3 ± 28.44	80.3 ± 46.13	42.6 ± 27.33	0.033
CRP (mg/dl)	35.5 ± 19.53	66.5 ± 35.43	30.5 ± 14.03	0.041
PLT (× 10^3^ /mm^3^)	404.89 ± 180.55	501.98 ± 123.21	305.87 ± 117.22	0.168
AST(U/l)	63 ± 121	69 ± 341	34 ± 202	0543
ALT(U/l)	51.9 ± 213	63.9 ± 235	37.9 ± 113	0.154
Alb(g/dl)	3.93 ± 0.44	3.21 ± 0.49	3.91 ± 0.44	0.233
Total Pro(g/dl)	5.7 ± 4.5	3.7 ± 4.1	5.9 ± 3.5	0.122
Bun(mg/dl)	25 ± 11	28 ± 10	24 ± 13	0.523
Cr(mg/dl)	0.5 ± 0.2	0.7 ± 0.4	0.5 ± 0.3	0.452

WBC: white Blood cell, Hb: Hemoglobin, CRP: C-reactive protein, PLT: platelets, AST: Aspartate transaminase, ALT: Alanine transaminase, Alb: albumin, Cr: Creatinine, BUN: blood urea nitrogen

Regarding laboratory findings, our study observed lymphopenia in 42% of patients, contrasting with a higher incidence of 64% in similar studies. Elevated inflammatory markers were detected in 72% of patients with CRP levels exceeding 50 mg/L and 24% with an ESR surpassing 40 mm/hour.

Pericardial effusion was the predominant cardiac involvement, identified in 75% of patients. Management of mild pericardial effusion, hypotension, or tachycardia included dexamethasone administered up to 3 mg every 8 h for 72 h. Additionally, aspirin at a dosage of 5 mg/kg daily, given once a day for two weeks, was prescribed. Follow-up echocardiography after two weeks revealed complete resolution of pericardial effusion in 100% of cases.

Nine patients exhibited coronary artery aneurysms. Of these, three patients saw resolution two weeks post-intravenous immunoglobulin (IVIG) treatment, while the remaining six experienced resolution after eight weeks following IVIG administration. Among the six patients with myocarditis, two suffered cardiac arrest due to heart failure and underwent cardiopulmonary resuscitation and intubation but, unfortunately, did not survive. Regarding the severity of symptoms, it appears that the disease spectrum varied among the patients, with some experiencing severe forms of the infection, particularly those with cardiac involvement, leading to conditions like myocarditis and coronary artery aneurysms.

## Discussion

During the COVID-19 pandemic, Multisystem Inflammatory Syndrome in Children (MIS-C) has been notably observed. This prospective study documented a significant surge in MIS-C cases in children, occurring approximately two months after the peak of infections in adults during the fifth wave of COVID-19 in Iran. In alignment with prior research, such as Alkan et al. and Yilmaz et al., we observed a male predominance (2.5:1) and noted the highest frequency of MIS-C in children aged 1 to 5 years, consistent with their findings [11, 12]. The age disparity observed in our study, with a mean age of 14 years in MIS-C cases, starkly contrasts the traditionally reported age range of 8–10 years [13]. This divergence highlights the imperative to reassess and broaden the age spectrum considered at risk for MIS-C, urging vigilance across pediatric age groups beyond the previously established boundaries.

The most prevalent symptom among patients was fever (74%), with a fever duration of more than five days considered a crucial indicator for MIS-C, particularly among those unresponsive to antibiotic treatment. Gastrointestinal symptoms, especially diarrhea (54%), were also prevalent in hospitalized patients, consistent with other studies [14–17].

In our study, lymphopenia was observed in 42% of patients, whereas Johnson et al. reported a higher incidence of 56% in their retrospective analysis, closer to our findings but slightly lower [18]. Conversely, Garcia et al.‘s prospective study found a significantly higher incidence of lymphopenia, at 68%, notably contrasting with our study and Johnson et al.‘s findings [19]. These variations might stem from differences in patient demographics, the timing of data collection relative to the disease course, or variations in diagnostic criteria used among the studies.

Regarding elevated inflammatory markers, our study noted CRP levels (> 50 mg/L) in 72% of patients, consistent with Johnson et al.‘s findings of 70%. However, Garcia et al. reported a higher incidence of elevated CRP levels, with 80% of patients exceeding this threshold. Similarly, our observation of elevated ESR (> 40 mm/s) in 24% of patients aligns with Garcia et al.‘s study [19], where they reported a slightly lower incidence of 20%, while Johnson et al. [18] found a notably lower incidence at 18%. These discrepancies could result from variations in patient populations, differences in laboratory measurement techniques, or diverse disease severity among the studies. These comparisons underline the variability in lymphopenia and inflammatory marker profiles among different studies, highlighting the need for further investigation into the factors contributing to these discrepancies and their potential implications for understanding and managing Multisystem Inflammatory Syndrome in Children (MIS-C).

The evaluation via echocardiography in hospitalized patients revealed cardiac involvement in 81% of cases, with pericardial effusion being the predominant cardiac manifestation in 75% of patients. Considering this prevalence, the term “Post COVID-19 pericardial effusion” might be more appropriate than using the broader term MIS-C in such instances. Contrary to Kawasaki disease, where coronary artery involvement typically occurs in children under five years old, our study noted that patients with coronary artery involvement were predominantly older than five years [21].

Immunomodulatory therapy stands as the primary treatment for MIS-C. Consistent with recommendations from Toubiana et al., our approach involved treatment with Intravenous Immunoglobulin (IVIG) or glucocorticoids. In cases where pericardial effusion was identified, treatment with dexamethasone (up to 3 mg every 8 h for 72 h) and aspirin (5 mg/kg daily for two weeks) resulted in complete resolution of pericardial effusion in all patients during follow-up echocardiography [20, 21].

In our study, only 12% of patients required ICU care, aligning closely with the findings of Lee et al., who reported a comparable rate of 14% [22]. However, Roberts et al.‘s study found a notably higher ICU admission rate, with 65% of patients requiring intensive care [22], indicating a significant discrepancy compared to our research and Lee et al.‘s findings. These differences might stem from variations in the severity of cases in each study, differences in healthcare systems or protocols, or variations in patient demographics.

Furthermore, among those requiring ICU care, our study documented two patients needing intubation, which is consistent with Lee et al.‘s findings of a similar intubation rate [21]. However, Roberts et al.‘s study reported a higher proportion of patients requiring intubation within the ICU, with 40% needing this intervention. Notably, unfortunately, one patient in our study succumbed to the condition during their ICU stay, which aligns with the outcomes reported by Lee et al. as they also reported a mortality rate consistent with our findings. However, the study by Roberts et al. reported a significantly lower mortality rate within their ICU cohort [23].

In our study, the cause of mortality in both patients was cardiac involvement. Among the six patients with myocarditis, two suffered cardiac arrest due to heart failure and underwent cardiopulmonary resuscitation and intubation but did not survive. In the study of Lorena Acevedo et al., all the patients who died had cardiac complications [24]. The severity of myocardial involvement, arrhythmias, and shock at the time of admission, along with underdiagnosis, may have contributed to these worse outcomes. To make a rapid and adequate diagnosis, the early initiation of anti-inflammatory treatment, when indicated, can significantly improve patient outcomes.

These comparisons highlight the variability in ICU admission rates, intubation needs, and mortality among studies investigating Multisystem Inflammatory Syndrome in Children (MIS-C). Further analysis is necessary to understand the underlying factors contributing to these discrepancies and their implications for clinical management and prognostication of MIS-C.

Furthermore, although the study did not investigate COVID-19 antibodies, this information underscores the potential association between recent COVID-19 exposure or infection and the subsequent development of MIS-C. Additionally, the absence of vaccination among these affected children highlights the scenario of susceptibility to MIS-C among unvaccinated individuals.

## Conclusions

Multisystem Inflammatory Syndrome in Children (MIS-C) manifests with a diverse array of clinical symptoms. Clinical suspicion is vital among physicians to promptly diagnose this syndrome, particularly in prolonged fever accompanied by gastrointestinal symptoms. Notably, most cases of pericardial effusion were solitary and demonstrated improvement following glucocorticosteroid treatment. This study highlights the importance of recognizing and appropriately managing cardiac involvement in the context of MIS-C associated with post-COVID-19 complications.

## Data Availability

All data generated or analyzed during this study are included in this published article.
